# Progress towards malaria elimination in the Greater Mekong Subregion: perspectives from the World Health Organization

**DOI:** 10.1186/s12936-024-04851-z

**Published:** 2024-03-01

**Authors:** Giulia Manzoni, Rady Try, Jean Olivier Guintran, Céline Christiansen-Jucht, Elodie Jacoby, Siv Sovannaroth, Zaixing Zhang, Vilasack Banouvong, Matthew Scott Shortus, Rita Reyburn, Chitsavang Chanthavisouk, Nay Yi Yi Linn, Badri Thapa, San Kyawt Khine, Prayuth Sudathip, Deyer Gopinath, Nguyen Quang Thieu, Mya Sapal Ngon, Dai Tran Cong, Liu Hui, James Kelley, Neena Nee Kesar Valecha, Maria Dorina Bustos, Charlotte Rasmussen, Luciano Tuseo

**Affiliations:** 1 WHO Mekong Malaria Elimination Programme, Phnom Penh, Cambodia; 2 World Health Organization Country Office, Phnom Penh, Cambodia; 3 National Center for Parasitology, Entomology and Malaria Control, Phnom Penh, Cambodia; 4 Centre for Malariology, Parasitology and Entomology, Vientiane, Lao PDR; 5 World Health Organization Country Office, Vientiane, Lao PDR; 6 National Malaria Control Programme, Nay Pyi Taw, Myanmar; 7 World Health Organization Country Office, Yangon, Myanmar; 8 Division of Vector Borne Diseases, Department of Disease Control, Bangkok, Thailand; 9 World Health Organization Country Office, Bangkok, Thailand; 10 National Institute of Malariology, Parasitology and Entomology, Hanoi, Viet Nam; 11 World Health Organization, Hanoi, Viet Nam; 12 Yunnan Institute of Parasitic Diseases, Yunnan, China; 13 World Health Organization, Regional Office for the Western Pacific, Manila, Philippines; 14 World Health Organization, Regional Office for South-East Asia, New Delhi, India; 15 Global Malaria Programme, World Health Organization, Geneva, Switzerland; 16 Present Address: Independent Consultant, Antananarivo, Madagascar; 17 Present Address: Independent Consultant, Le Bar sur Loup, France; 18 Present Address: Independent Consultant, Ho Chi Minh, Viet Nam

**Keywords:** Antimalarial drug resistance, Malaria, Elimination, World Health Organization, Greater Mekong Subregion

## Abstract

Malaria remains a global health challenge, disproportionately affecting vulnerable communities. Despite substantial progress, the emergence of anti-malarial drug resistance poses a constant threat. The Greater Mekong Subregion (GMS), which includes Cambodia, China’s Yunnan province, Lao People's Democratic Republic, Myanmar, Thailand, and Viet Nam has been the epicentre for the emergence of resistance to successive generations of anti-malarial therapies. From the perspective of the World Health Organization (WHO), this article considers the collaborative efforts in the GMS, to contain *Plasmodium falciparum* artemisinin partial resistance and multi-drug resistance and to advance malaria elimination. The emergence of artemisinin partial resistance in the GMS necessitated urgent action and regional collaboration resulting in the Strategy for Malaria Elimination in the Greater Mekong Subregion (2015–2030), advocating for accelerated malaria elimination interventions tailored to country needs, co-ordinated and supported by the WHO Mekong malaria elimination programme. The strategy has delivered substantial reductions in malaria across all GMS countries, with a 77% reduction in malaria cases and a 97% reduction in malaria deaths across the GMS between 2012 and 2022. Notably, China was certified malaria-free by WHO in 2021. Countries' ownership and accountability have been pivotal, with each GMS country outlining its priorities in strategic and annual work plans. The development of strong networks for anti-malarial drug resistance surveillance and epidemiological surveillance was essential. Harmonization of policies and guidelines enhanced collaboration, ensuring that activities were driven by evidence. Challenges persist, particularly in Myanmar, where security concerns have limited recent progress, though an intensification and acceleration plan aims to regain momentum. Barriers to implementation can slow progress and continuing innovation is needed. Accessing mobile and migrant populations is key to addressing remaining transmission foci, requiring effective cross-border collaboration. In conclusion, the GMS has made significant progress towards malaria elimination, particularly in the east where several countries are close to *P. falciparum* elimination. New and persisting challenges require sustained efforts and continued close collaboration. The GMS countries have repeatedly risen to every obstacle presented, and now is the time to re-double efforts and achieve the 2030 goal of malaria elimination for the region.

## Background

Malaria continues to inflict a heavy burden worldwide, perpetuating a cycle of ill health and poverty that disproportionally affects communities with limited access to healthcare and resources. Although there has been considerable progress in reducing malaria-related deaths and cases, combating this parasitic disease remains a formidable challenge [1]. In particular, the emergence and spread of resistance to critically important anti-malarial drugs are a constant threat which must be addressed through strong collaborative efforts, innovative solutions and a commitment to malaria elimination.

**Fig. 1:**
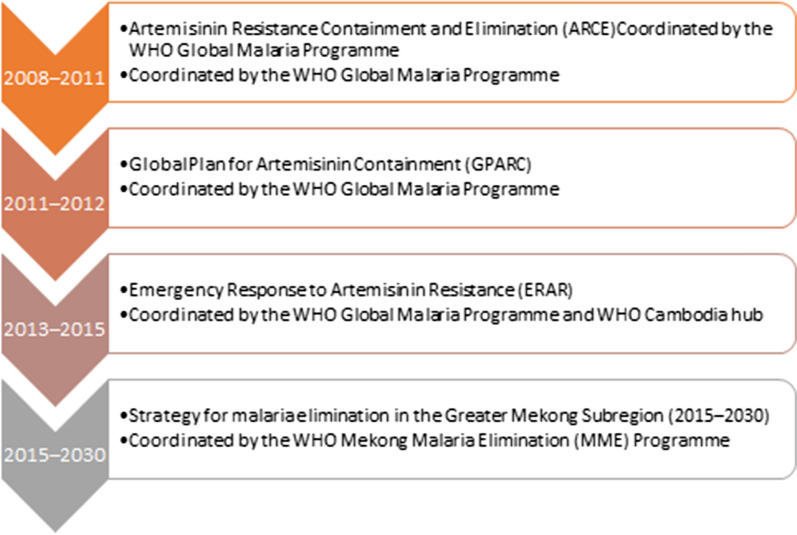
Evolving strategies to address artemisinin partial resistance and multi-drug resistance

This article considers the regional response of the Greater Mekong Subregion (GMS) countries to the emergence of *Plasmodium falciparum* parasites expressing partial resistance to artemisinin and multi-drug resistance from the perspective of the World Health Organization (WHO). Since 2008, and co-ordinated through various initiatives, the WHO has supported drug resistance containment and malaria elimination activities across the six GMS countries—Cambodia, China (Yunnan Province), the Lao People’s Democratic Republic (PDR), Myanmar, Thailand, and Viet Nam. The region aims to eliminate *P. falciparum* by 2025 and all human malaria by 2030 (Fig. 1).

Drawing on epidemiological data, published literature, the WHO and partner meetings reports, country reports and case studies, this perspective considers the challenges encountered and witnesses the accomplishments, progress and cooperation achieved by the GMS countries in addressing malaria across the region.

## Anti-malarial drug resistance in the GMS: a constant challenge

Historically, the GMS has been a hot-spot for the emergence of anti-malarial drug resistance. Chloroquine-resistant *P. falciparum* first emerged in Cambodia in the late 1950s, and within 30 years this once potent drug was clinically ineffective across malaria endemic regions globally [2]. Similarly, sulfadoxine–pyrimethamine resistance emerged in the GMS soon after its introduction and rapidly spread to Africa [3]. These experiences illustrated the need for vigilant monitoring and adaptive responses to prevent history from repeating itself with other anti-malarial drugs.

The discovery of artemisinin in China introduced a new class of drugs with fast and potent anti-malarial activity [4, 5]. In the GMS, artemisinin was initially deployed as monotherapy, but its effectiveness was limited by the need for a 7-day treatment regimen [5–7]. Inadequate dosing and poor adherence led to high recrudescence rates [5–7]. The solution was to combine artemisinin with a longer-acting partner drug from a different drug class, resulting in a highly effective three-day dosing schedule. Combination therapy also theoretically protects against resistance selection for both components [8]. By 2006, artemisinin-based combination therapy (ACT) became the recommended global first-line treatment for uncomplicated *P. falciparum* malaria, and today, ACT is the cornerstone of malaria treatment [1].

The emergence of *P. falciparum* parasites demonstrating partial resistance to artemisinin was first reported in Battambang Province in western Cambodia in 2008 [9], and further confirmed in Pailin Province in 2009 [10]. The reduced sensitivity of early intraerythrocytic parasite stages to artemisinin was characterized phenotypically by slow parasite clearance [11]. Various non-synonymous mutations in the propeller region of *P. falciparum kelch 13* gene were validated as molecular markers for partial artemisinin resistance, enabling detailed molecular surveillance of its evolution and spread across the GMS [12].

Artemisinin partial resistance does not affect ACT clinical efficacy at Day 28, but it is associated with persistent parasitaemia at Day 3 [13]. This leaves the partner drug exposed to greater numbers of parasites and hence a higher risk of resistance selection [14]. Soon after the introduction of dihydroartemisinin–piperaquine and mefloquine-artesunate high clinical failure rates were reported from several areas of the GMS [15, 16]. The validation of molecular markers for piperaquine and mefloquine resistance allowed the spread of multi-resistant parasites to be tracked [17].

The dissemination of multi-drug resistance across the GMS threatened to make malaria untreatable in the region. Like chloroquine resistance, it was feared that multi-drug resistant parasites would be transported internationally. Potentially, this would lead to the end of ACT as an effective therapy for uncomplicated malaria worldwide. With no new anti-malarial drug classes available, it was clear that artemisinin partial resistance had to be contained within the GMS and that the only solution to address multi-drug resistance was malaria elimination. This great responsibility fell upon the countries of the GMS, specifically their National Malaria Programmes (NMPs).

## Coordinated strategies for drug resistance containment and elimination

Strategies and approaches for containing drug resistance in the GMS have evolved with the changing epidemiological situation and evidence (Fig. 1). The initial focus was on containing artemisinin partial resistant parasites in Cambodia and Thailand through the Artemisinin Resistance Containment and Elimination (ARCE) project (2008–2010), funded by the Bill and Melinda Gates Foundation (BMGF) and coordinated by the WHO Global Malaria Programme [18–20]. Key outcomes of ARCE were the banning of artemisinin monotherapy by the Cambodian Ministry of Health in 2009, and the implementation of the electronic Malaria Information System by the Thailand Bureau of Vector-borne Diseases. This provided near real-time data for case detection, investigation and follow-up, with identification of potential drug resistance along the Thailand–Cambodia and Thailand–Myanmar borders [18, 21]. Although ARCE was not able to contain artemisinin partial resistance within Cambodia and Thailand, it showed that intensive surveillance, aggressive interventions to combat malaria, and collaboration across borders could significantly reduce the malaria burden [18, 19, 21]. Crucially, ARCE provided evidence that malaria elimination was an achievable goal in the GMS [18, 19, 21].

The risk that artemisinin partial resistance could not be checked in the GMS was recognized in the Global Plan for Artemisinin Containment (GPARC) which aimed to increase vigilance and rapidly respond to the issue worldwide [22]. To contain multi-drug resistant parasites within the GMS, the WHO Emergency Response to Artemisinin Resistance (ERAR) was launched in 2013, including all six GMS countries and targeting malaria elimination [23]. Aims included improving access to malaria prevention, diagnostics and treatment, the establishment and strengthening of surveillance systems across the region to allow more timely detection of drug resistance, prioritization of research goals, and co-ordination of operational research to inform policy [24, 25]. Between 2012 and 2015, malaria incidence across the GMS was reduced by more than 54% and deaths by 84%, indicating that these interventions were creating impact [26].

Following the lead of the ERAR, the Regional Artemisinin-resistance Initiative (RAI) of the Global Fund to Fight AIDS, Tuberculosis and Malaria (Global Fund) was established for 2014–2017 to support malaria elimination in Cambodia, Lao PDR, Myanmar, Thailand, and Viet Nam. Further RAI grants were made for 2018–2020, 2021–2023, and 2024–2026 coordinated by the RAI Regional Steering Committee. Activities have focused on strengthening national surveillance, increasing access to malaria services in remote populations in border regions, and case management through supporting the recruitment and training of community health volunteers [27].

The insights gained from ARCE and ERAR provided the foundations for the WHO Strategy for Malaria Elimination in the Greater Mekong Subregion (2015–2030) [28]. Developed after a wide consultation process, including GMS countries’ NMPs and partner organizations, the strategy aimed for an accelerated scale-up of appropriate interventions tailored to the local epidemiology, with prioritization of malaria elimination in areas with multi-drug resistance, transmission reduction in high transmission areas as well as preventing and responding to malaria resurgence. To support these goals, the WHO Mekong Malaria Elimination (MME) programme was established in 2017, funded by RAI and the BMGF. Headquartered in Phnom Penh, Cambodia, MME collaborates closely with various WHO offices, NMPs, and partners to promote dialogue, strengthen surveillance systems, accelerate elimination, provide technical support for implementation, disseminate guidance on malaria control, and facilitate the malaria elimination certification process. The strategy has successfully checked the spread of multi-drug resistance in the GMS, ensured the continued efficacy of anti-malarial therapy in every GMS country, and driven substantial progress towards malaria elimination.

## Country ownership, strategic responsibilities and accountability

The goal of malaria elimination in the GMS was endorsed in 2018 by the Ministers of Health across all the GMS countries [28]. This high-level political support has been crucial in supporting the national and regional elimination programme.

To identify priorities and funding requirements, each country creates an annual work plan with the support from MME, integrated into the national strategic plan. Plans need to be evidence-based, feasible, with agreed outcomes that benefit communities and allow donors to monitor their investment. Activities are prioritized based on their expected impact on malaria elimination, feasibility, capacity, potential for funding, political support, and urgency. For example, early prioritization exercises led to the banning of artemisinin monotherapy in the private sector in Cambodia, and strengthening of surveillance in Lao PDR, Myanmar, Thailand, and Viet Nam; all of these having a significant impact on malaria elimination in the GMS. This process encourages consistent evaluation of programmatic interventions and leads to improvements in programme effectiveness and understanding of interventions. NMPs conduct a mid-term plan review to allow re-adjustment to accommodate any changes as required.

Each year, MME organizes regional workshops on WHO policy guidance, surveillance, elimination, and therapeutic efficacy studies (TES), which also includes integrated drug efficacy surveillance (iDES). These meetings provide a forum for GMS countries to share their learnings, present their plans, and get feedback and insights from other countries’ NMP representatives, partners, and donors. It also allows the WHO to communicate policy revisions based on recommendations from the Global Malaria Programme. Conversely, perspectives shared by GMS countries have informed the updated WHO guidelines for malaria, which include recommendations for malaria elimination intensification, acceleration, and reactive activities with flexibility for programmes to tailor interventions to their requirements [1].

Countries regularly run national and sub-national training workshops on data management, microscopy, laboratory techniques, TES and iDES implementation, and malaria elimination with technical assistance from MME and WHO country offices, where needed. Additionally, countries are responsible for training, monitoring and incentivizing thousands of community-based village malaria workers (VMWs). This has led to innovations, such as national awards and local incentive schemes that empower communities and increase the opportunities for learning across the GMS. It is also an increasingly sustainable approach as training capacity is strengthened within countries.

## Progress towards malaria elimination in the GMS

**Fig. 2:**
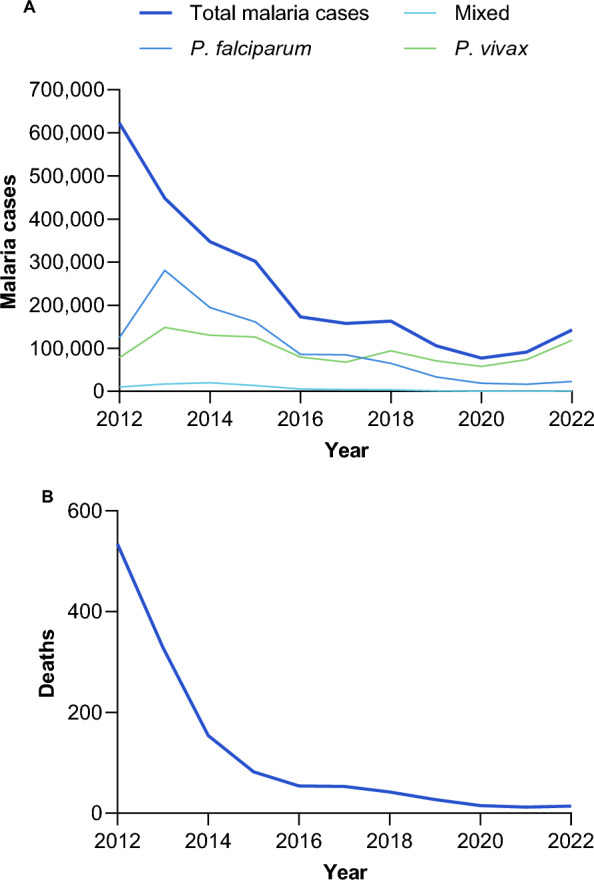
Incidence across the GMS of **A** malaria cases and **B** malaria deaths (Source: MEDB database)

In the last decade (2012–2022) there has been a rapid and sustained decline in malaria cases and deaths across the GMS, though cases increased in 2022, mainly because of a resurgence of malaria in Myanmar, and spill-over of cases across the border into Thailand (Fig. 2).

**Fig. 3:**
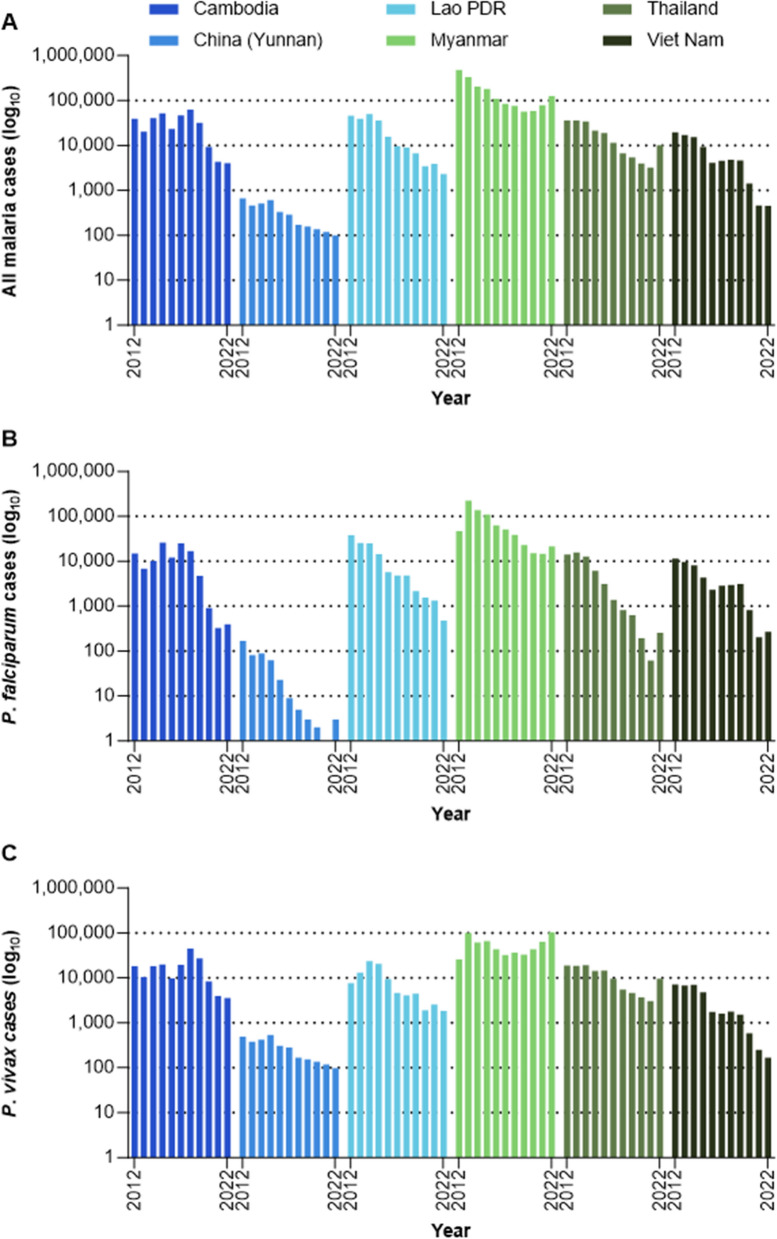
Malaria case incidence by country for **A** overall, **B** *P. falciparum* and **C** *P. vivax*. Note the log_10_ scale, i.e., each dotted horizontal line represents and tenfold change in incidence (Source: MEDB database)

In 2012, *P. falciparum* caused 60% of the approximately 641,000 reported malaria cases in the GMS and, except for Yunnan Province in China, all the GMS countries reported more than 10,000 *P. falciparum* cases per year (Fig. 3). With the goal of eliminating *P. falciparum* by 2025, innovative strategies were adopted across the GMS to accelerate *P. falciparum* elimination in the region. These included high quality case management and investigation with real-time epidemiological surveillance to the individual case level, drug resistance surveillance, decentralization of malaria services to the community level, increased testing capacity, and targeted chemoprevention. By 2022, these interventions had delivered a 77% reduction in malaria cases overall and a 94% reduction in *P. falciparum* cases and by 2022, cases were below 500 per year in China, Thailand, Vietnam, Cambodia, and Lao PDR (Fig. 3). Remaining challenges to *P. falciparum* elimination are the continued threat of drug resistance, addressing foci in difficult to access and mobile populations, and maintaining malaria surveillance, control and treatment services against declining transmission.

Between 2012 and 2022, *P. vivax* cases declined by 48% and by 2017, *P. vivax* had overtaken *P. falciparum* as the predominant cause of malaria in the GMS. In 2022, *P. vivax* accounted for 83% of all cases, with only China and Viet Nam having fewer than 1000 cases per year (Fig. 3). The primary challenge to *P. vivax* elimination is the ability of the parasite to form hypnozoites which lie dormant in the liver until reactivation weeks or months after the initial infectious bite. This increases the malaria burden and sustains the transmission reservoir by allowing the parasite to evade malaria control efforts. With the goal of eliminating all human malaria in the GMS by 2030, new strategies are needed to address *P. vivax* and countries are developing context specific plans to deploy new tools addressing the characteristics of vivax malaria. These include radical cure with 7-day primaquine or single-dose tafenoquine to prevent relapses incorporating point-of-care testing for glucose-6-phosphate dehydrogenase deficiency (G6PD), iDES with case follow-up to day 90 to detect relapses, and information education communication/behaviour change communication (IEC/BCC) activities to give high-risk populations the tools to reduce individual risk of infection.

## China malaria-free certification

The certification of malaria elimination in China in June 2021 was a major milestone for the GMS. China's success in achieving malaria elimination was attributed to political commitment, domestic funding, free access to affordable malaria diagnosis and treatment, effective multi-sector collaboration across thirteen government ministries, and implementation of the ‘1-3-7’ strategy [29–31]. This strategy requires the reporting of all malaria diagnoses within one day, case confirmation and risk assessment within three days, and appropriate measures to prevent further disease spread taken within seven days [30].

## Adapting malaria elimination strategies

**Fig. 4:**
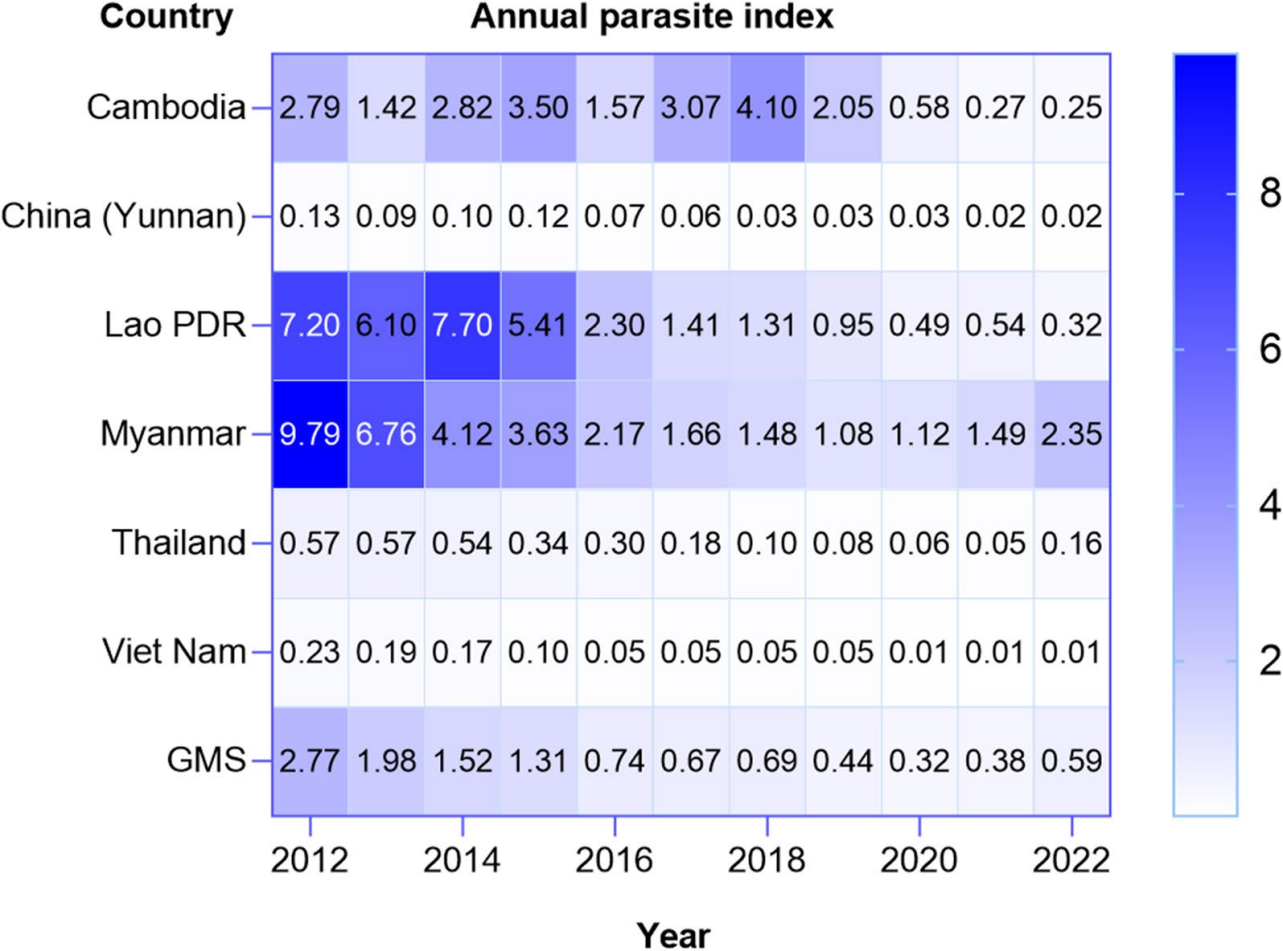
Annual parasite index has declined across all countries in the GMS. Annual parasite index is the number of confirmed new cases from malaria registered in a specific year, expressed per 1000 individuals under surveillance, for a given country, territory, or geographic area. Activities directed at malaria elimination are feasible where the API is below 1.0 [28]. (Source: MEDB database)

Strategies for malaria elimination necessarily evolve with the changing epidemiological situation. Since 2012, all the GMS countries have seen dramatic declines in their annual parasite index (API), i.e., the number of malaria cases per 100 people at risk per year, though there have been recent increases in Myanmar and Thailand (Fig. 4). According to the strategy for malaria elimination in the Greater Mekong Subregion: 2015–2030, activities should be focused on burden reduction in at-risk populations where the API is above 1.0. Below this threshold, malaria elimination is feasible, and activities include rigorous surveillance and management of active foci. GMS countries are at different points in their progress, with Myanmar focusing on burden reduction, Cambodia, Lao PDR, Thailand, and Viet Nam on malaria elimination, and China on prevention of re-establishment of malaria.

## Intensification strategies to reduce burden in Myanmar

Historically, Myanmar has borne the greatest burden of malaria in the GMS. Significant progress towards reducing the malaria burden was made until 2020, but cases began increasing in 2021 and 2022. This was caused by political instability interrupting the delivery of malaria services, particularly in remote border areas where malaria is most prevalent.

Although burden reduction is the primary goal in Myanmar, the picture is highly heterogeneous. In 2022, 275 townships with an API < 1 were implementing elimination activities, 186 townships reported no *P. vivax* or mixed cases, and 52 townships accounted for 68% of *P. falciparum*/mixed cases and 59% of *P. vivax* cases. To address this heterogeneity and effectively direct interventions, Myanmar undertook village level malaria risk stratification and, with support from the WHO, developed a targeted intensification and acceleration plan. In areas of high malaria risk, activities include passive case detection, targeted mass screen and treat, *P. vivax* radical cure, distribution of long-lasting insecticidal nets (LLINs), routine indoor residual spraying (IRS), and community-based IEC/BCC activities. Additionally, malaria case-based reporting is being reinforced, with mandatory case notification, classification, and investigation within 24 h, and case response completed within 7 days. These interventions are consistent with WHO guidelines and have been shown to be effective in reducing malaria burden and preparing for transition to the elimination phase [1, 32–39].

## Acceleration to elimination strategies in Cambodia, Lao PDR, Thailand, and Viet Nam

Following successful intensification to reduce malaria burden, and applying the lessons learned from these approaches, Cambodia, Lao PDR, Thailand, and Viet Nam are seeking out and aggressively targeting the remaining pockets of transmission [1, 40–43]. The aim is to investigate and manage every case effectively and take steps to interrupt transmission in active foci through appropriate vector control and anti-malarial drug-based interventions.

To further accelerate malaria elimination, Cambodia and Lao PDR are implementing ‘last-mile to malaria elimination’ strategies [41, 44–49]. These strategies aim to decentralize malaria services, transferring them from health centres to community-based delivery through the efforts of VMWs and mobile malaria workers. These workers take on expanded roles that include strengthening early diagnosis and treatment with passive and active case detection, reactive case detection, enhancing vector control, including distribution LLIHN, LLIN, and personal protection packs for forest goers. Additionally, VMWs implement preventive measures for vulnerable communities such as TDA and intermittent preventive treatment for high-risk groups, such as forest-goers (IPTf). In Lao PDR, additional high-risk groups were identified through community surveys, such as people who sleep in the field and teenagers who socialize outside late in the evening. To accept increased testing, regular fever screening and the adoption of chemoprevention, communities need to recognize VMWs as dependable providers of malaria care services. VMWs are invaluable in leveraging their local knowledge and expertise to engage and educate households about malaria risks, elimination goals, and the need to abide strictly by the full and correct regimen of preventive measures such as TDA and IPTf.

Another approach to support acceptance of acceleration initiatives has been the application of ethnographic research [48]. Studies conducted by NMPs revealed linguistic, cultural, and behavioural variations among communities affecting malaria transmission, especially in high-risk zones like forests and cultivation fields. This diversity can be a significant challenge to successful uptake of interventions, potentially compromising elimination progress. For example, house-to-house visits used to reinforce the surveillance system and ensure early case management were effective in some areas while considered unacceptable in others due to cultural beliefs. This high level of heterogeneity underlines the importance of delivering malaria services within communities by community members.

Supporting and reflecting the need to de-centralize malaria services, the MME programme deploys technical staff to where malaria transmission is prevalent. Employing a problem-solving approach, WHO epidemiologists and NMP central and local staff work closely with provincial and district malaria supervisors and partners to enhance coordination among stakeholders and align malaria activities with community census and epidemiological and mapping analyses. Weekly ad-hoc field visits are organized at hospitals, health facilities, and in villages to identify and address gaps and challenges while monitoring implementation quality. The dialogue between the provincial and central levels is strengthened through regular joint supervision visits and monthly technical working groups convened at both peripheral and central levels by NMPs. By actively participating in the implementation of malaria activities, all partners gain a thorough understanding of the malaria situation, generating context-appropriate strategies.

## Prevention of reestablishment of malaria in China

China remains highly receptive to malaria transmission, with a widespread distribution of *P. vivax* vectors. There were 1170 malaria cases imported from six bordering countries between 2017 and 2021, nearly all of which (98.4%) were *P. vivax* [50]. In 2022, of 94,942 suspected malaria cases tested in China, 101 were confirmed, with 95 classified as imported and six unclassified. As well as requiring continued surveillance and effective case management and investigation within China, the country depends on its neighbours to address outbreaks and foci that might jeopardize its malaria-free status [50, 51]. For example, a joint reporting system was established with China, Lao PDR and Myanmar to report malaria cases along the international border, malaria checkpoints were established at key locations to provide screening and malaria services to mobile and migrant populations (MMPs), and the health system was strengthened in counties along the borders with Lao PDR and Myanmar. Community education to maintain awareness of malaria risk in areas prone to importation is also key so that treatment is sought promptly and transmission prevented [51].

The continued risk of imported malaria from Chinese citizens returning from malaria endemic regions such as sub-Saharan Africa, remains a significant concern [52]. Thus, strengthening malaria surveillance in high-risk areas and continuing to actively engage in regional malaria control initiatives are crucial for China. The establishment and maintenance of such a widespread and responsive surveillance system requires continued vigilance within the affected communities, multi-sectorial co-ordination, strong political support and effective cross-border collaboration.

## Regional challenges to malaria elimination in the GMS

There are two overarching challenges to delivering effective malaria elimination strategies across the GMS. Firstly, malaria transmission primarily occurs in forested areas most often located around the border areas between GMS countries, necessitating effective cross-border collaboration to contain or eliminate the disease [37, 53–57]. Secondly, MMPs are at highest risk of contracting malaria, as these populations often live in remote and hard-to-access areas, face social barriers like discrimination, and may be undocumented or engaged in illegal activities, making it difficult to reach and provide effective malaria services [33, 58–60].

## Cross-border collaboration

From the start, it was recognized that cross-border collaboration would be critical in any attempts to contain or eliminate malaria in the GMS [54, 56, 61–65]. A pioneering collaboration during the time of ARCE involved joint action by Cambodia and Thailand to strengthen surveillance systems, ensure prompt and effective treatment with ACT, enhance vector control measures, and support community mobilization through IEC/BCC, with harmonized messages targeting MMPs [18, 21]. Additionally, cross-border technical workshops directed primarily at NMPs promoted coordination of activities. These activities generated evidence to inform elimination across the GMS as well as fostering ties between engaged countries [18, 21]. Malaria control was incorporated into the Thailand–Cambodia Development Cooperation Programme for Health (2017–2020 and 2021–2023), enhancing the standardized control of communicable diseases through joint training on surveillance and emergency response, and establishing strong communication channels between Thai and Cambodian health staff.

By 2011, China, Myanmar, Vietnam, and Lao PDR had agreed to join Cambodia and Thailand to present a regional response to address the threat of artemisinin partial resistance across the GMS, with support from the WHO South-East Asia and Western Pacific offices. This allowed the WHO to work with partners and NMPs across the region to formulate and launch the ERAR [23], develop the strategy for malaria elimination in the GMS [28] and establish the MME programme. The structure of the MME programme has proved to be robust to changing political situations and the COVID-19 pandemic during 2020–2021, with cooperation and information sharing maintained. This is possible because of the long-standing collaborative relationships and trust that have been developed between the GMS countries in their shared goal of malaria elimination.

Countries that share borders have also developed cross-border programmes with their neighbours. In these agreements, rather than funding individual activities or projects, a comprehensive and integrated strategy for securing financial resources is adopted to provide resilient and sustainable funding for the whole range of activities required to reduce cases and effectively interrupt malaria transmission. For example, Cambodia, Thailand, and Myanmar have identified cross-border malaria elimination zones where collaborative efforts are focused, including the harmonization of IEC/BCC messages for target populations and collaborative improvement of tools and channels to reach remote or illiterate populations. Similarly, an integrated approach to collaboration dramatically decreased the malaria burden around the Chinese–Myanmar border and was a key contributor to China achieving malaria-free status [35, 66, 67].

As elimination is achieved in border regions, the risk of malaria importation must be addressed, with joint stringent surveillance systems and accurate classification of cases to identify imported cases versus re-establishment of local transmission. For example, in 2022 Viet Nam noted an increase in *P. vivax* cases in one district bordering Yunnan and is strengthening malaria services in the region, which may include the roll-out of targeted drug administration (TDA). This underlines the importance of maintaining close collaboration across borders as well as the interdependency of the GMS countries in achieving and maintaining their elimination goals.

## Mobile and migrant populations

A key aim of ERAR and the MME programme has been to address malaria in MMP populations [68]. MMPs do not represent a single group of people but many overlapping populations, such as refugees, forest workers, ethnic minorities, military, etc., who may be challenging to reach geographically, often living in remote hard-to-access locations. There are also social and cultural barriers, such as isolation and discrimination, lack of integration into local communities and different languages [33, 58–60]. They may also be undocumented or engaged in potentially illegal logging/mining activities and are often keen to avoid official channels.

To address this challenge, NMPs have identified the populations at risk, surveyed their needs and patterns of behaviour and developed targeted tailored strategies to provide effective and appropriate malaria services. This requires significant innovations and adaptations in systems and processes specific to each country with a focus on vector control and chemoprevention. For example, vector control measures, such as insecticide-treated mosquito nets (ITNs), repellent, or long-sleeved clothes showed limited effectiveness due to the forest workers’ frequent mobility, behaviour, and extended working hours [69–71]. To better meet the needs of MMPs Viet Nam established community malaria action teams in 86 at-risk communities, responsible for distributing long-lasting insecticidal hammock nets (LLIHNs), conducting surveillance, case referral, and tailored IEC/BCC activities. In very remote areas, mobile outreach teams are deployed to strengthen active case detection and treatment, engage with the community and improve the capacity for malaria epidemiological surveillance. In China, migrants from frontier townships are regularly screened for malaria and treated if necessary to prevent malaria re-establishment. Similarly, Thailand conducts active case detection in low transmission areas where there is an influx of new migrants, such as seasonal workers.

## Surveillance, monitoring and evaluation

The task of the NMPs would not be possible without high quality surveillance data and surveillance strengthening has been a core activity supported by sustained funding from RAI, BMGF and the U.S. Agency for International Development President’s Malaria Initiative (USAID-PMI). NMPs must continually verify monitor case reporting, treatment efficacy and rapidly instigate changes to treatment policy if indicated, make decisions on the appropriateness of interventions, and evaluate their effectiveness, ensure efficient deployment of limited resources, quickly identify and address malaria outbreaks, monitor malaria importation, and prevent the re-establishment of transmission.

## Drug resistance surveillance

Anti-malarial drug resistance surveillance to ensure drug efficacy, inform treatment policies, and track resistance emergence and spread. Since 2008, TES have provided key evidence supporting national treatment guidelines. In consultation with NMPs and principal investigators, the WHO developed a standard TES protocol and offered technical support in establishing sentinel sites across the GMS, with monitoring visits to assess implementation [72]. Ethical approval for TES studies was available through the WHO Ethical Review Committee (ERC) or the WHO Regional ERC, plus national ERCs. In collaboration with partners, the laboratory network was strengthened and in-country training workshops held for field staff and microscopists, with monitoring visits to assess implementation [72]. Standard operating procedures to differentiate *P. falciparum* recrudescence and reinfection were developed. Also, countries adopted standard definitions of artemisinin partial resistance and multi-drug resistance [73]. The GMS TES network was established to share data across the GMS countries and foster collaboration. TES and were crucial in providing clear evidence of *P. falciparum* delayed parasite clearance with ACT and the emergence of piperaquine resistance in western Cambodia and mefloquine resistance in Thailand. In 2012, the indisputable data generated by these studies supported the Association of the South-East Asian Nations (ASEAN) Secretariat, NMPs, development partners and WHO to raise high-level political support to address this problem from a regional perspective and enabled formulation of the ERAR.

As the malaria burden has declined in the GMS, finding adequate case numbers within the required timeframe to meet sample size requirements for TES has become challenging in some countries. To address the need for continuing surveillance of the parasitological response to treatment in an elimination setting, iDES is being implemented in Cambodia, Lao PDR, Thailand, and Viet Nam. This approach uses routine data collected for malaria case management to generate information about drug effectiveness. Key to this approach is the availability of quality assured laboratory services and sophisticated data management to compile actionable information: in Thailand, iDES has captured data of sufficient quality to inform changes in malaria drug treatment policy [74]. The WHO continues to provide technical assistance in TES and iDES quality implementation and provides guidance on national and regional drug policy reviews.

A key challenge for iDES is that surveillance must include all malaria cases, and Cambodia, Myanmar, Lao PDR, Thailand, and Viet Nam have a significant private sector [75]. Engaging the private sector in surveillance activities requires training, provision of free diagnostics and ideally of first-line anti-malarial drugs also, and supportive supervision [76]. In addition, the private sector must have access to user-friendly reporting systems and must be incentivized to complete malaria case management and follow-up accurately and comprehensively. However, these investments are critical as private sector collaboration is a crucial component of the malaria-free certification process and will be a key element in the prevention of re-establishment of malaria.

The validation of molecular markers for artemisinin partial resistance, piperaquine and mefloquine resistance in the GMS has generated a thorough understanding of the evolution of parasite resistance in the region [17, 77]. Initially, it was thought that western Cambodia was the sole source of artemisinin partial resistance, but, molecular surveillance of genes associated with resistance showed multiple origins across the region with distinct genetic evolutionary pathways [73]. These findings of multiple instances of de novo resistance emergence heightened the need for surveillance strengthening across the GMS and elsewhere. For this reason, molecular surveillance was incorporated in both TES and iDES protocols to provide a greater understanding and faster recording of any changes in drug efficacy, with these data included in the WHO Malaria Threat Maps [78]. Additionally, WHO is collaborating with GenRe-Mekong (*Genetic* Re*connaissance of Malaria in the Greater* Mekong *Subregion*) which is funded by BMGF and aims to provide NMPs and other partner organizations with actionable genetic surveillance data.

## Epidemiological surveillance

Epidemiological surveillance to examine trends in malaria cases, transmission rates, and risk factors, usually involving data analysis, and mapping to identify high-risk areas and target interventions effectively.

A central component of the MME programme is the Malaria Elimination Database (MEDB), which collects and shares summaries of malaria data monthly and quarterly, in addition to the yearly MME Bulletin. Developing high quality reporting has required a commitment from countries in strengthening their data collection procedures and implementing technical data management services. Although countries have taken different approaches, all provide a minimum standard dataset to the MEDB and have agreed on common terminology and definitions. Reporting practices continue to evolve with all countries providing a monthly data set, including case management, with those targeting elimination also including case investigation (Table 1). The next priorities for MEDB development are the incorporation of case-based reporting for Viet Nam and strengthening the national surveillance databases in Myanmar, including integration to a single data repository. Additionally, training and data sharing activities will continue as well as technical improvements to streamline data collection and analysis.

**Table 1:** Surveillance data captured in the Malaria Elimination Database (MEDB) in 2022

Country	Case management	Elimination data		Reporting frequency	Disaggregated by age group and gender	Disaggregated by sector (health facility/community)	Private sector data included	Lowest level of data included	
		Case investigation	Foci investigation					National MIS	MEDB
Cambodia	✓	✓	✗	Monthly	✓	✓	NA^a^	Village	Health facility
China (Yunnan)	✓	✓	✗	Monthly	✗	✗	NA^b^	Village	County
Lao PDR	✓	✓	✓	Monthly	✓	✓	✓	Village	Health facility
Myanmar	✓	✗	✗	Monthly	✓	✗	✓	Village	Township
Thailand	✓	✓	✗	Monthly	✓	✗	Limited^c^	Village	Subdistrict
Viet Nam	✓	✗	✗	Monthly	✓	✗	✗	Commune	District

^a^Ministry of Heath deactivated the private sector in June 2018

^b^Private sector is not permitted to prescribe anti-malarial drugs

^c^Only some private hospitals

MEDB data have been used to track progress towards malaria elimination, support communication with donors and the wider scientific community, identify specific challenges, and importantly, enable greater opportunities for evidence-based cross border collaboration. The advancements in the MEDB have closely paralleled improved reporting mechanisms adopted by countries through their Malaria Information Systems and mHealth initiatives.

Within the countries, the primary objective of the epidemiological surveillance system is to furnish real-time information to all tiers of the healthcare system. For malaria control, surveillance is used to plan and evaluate national and sub-national activities, whereas in an elimination setting, the goal is to find each case and foci, requiring a high degree of data granularity. Development of an elimination-capable surveillance system demands significant investment in staff recruitment and training, communication infrastructure, computers, and enhanced software capabilities. Such investments are critical due to the high levels of heterogeneity in malaria transmission within the GMS, given that countries must simultaneously execute strategies for malaria control, pre-elimination, elimination, and prevention of re-establishment of transmission, as well as detecting outbreaks and directing responses.

Case and foci investigation need to be strengthened as countries approach elimination. In the GMS, China, Cambodia, Lao PDR, and Thailand have adopted the 1-3-7 approach [79–81]. This requires cases to be reported within one day of diagnosis, investigated within three days of reporting, with foci investigation and response, including vector control and reactive case detection, completed within seven days [82]. Viet Nam has implemented a 2-7 approach, with foci investigation within two days and foci response within seven days [83]. In Myanmar, a 1-7 timescale is used with case reporting within one day, and foci investigation and response within seven days [84]. Adherence to the timings is key, particularly in the case of *P. vivax* malaria where onward transmission to the mosquito has likely already occurred by the time a case has been identified. Thus, prompt reporting and foci investigation are needed to identify secondary infections and stop further transmission. Such intensive efforts can only be applied in areas of low malaria transmission. However, with sufficient resources this approach is feasible in an elimination setting, with very high adherence rates reported in China (> 98%) [85] and Cambodia (100%) [81]. Notably, where these approaches have been robustly implemented with high adherence, malaria case incidence has dramatically declined [82].

To prepare for malaria elimination and maintain malaria-free certification all countries must ensure that a robust surveillance system is in place to rapidly identify and respond to imported malaria cases. Hence, all countries in the GMS except Myanmar now aim to classify indigenous versus imported cases. In 2022, a classification of imported malaria was made for 36.0% of cases in Thailand, 2.8% in Lao PDR, 1.8% in Viet Nam and 0.1% in Cambodia.

## Entomological surveillance

Effective vector control is an essential component of malaria elimination [28]. Entomological surveillance involves the systematic collection, analysis, and interpretation of entomological data to assess risk, and optimize vector control interventions [86]. Key objectives include characterizing receptivity, tracking vector species density, and determining seasonality [86]. Additionally, insecticide resistance monitoring, identification of threats to vector control effectiveness, and assessing intervention coverage and quality are required [86].

The example of China illustrates how vector control and surveillance have been applied to malaria control, malaria elimination, and post-elimination [87]. National and provisional vector surveillance sites were established with annual surveys of the vector population and density, and sentinel sites to monitor vector insecticide resistance [87]. An understanding of vector distribution and biology informed targeted vector control approaches, such as IRS of animal enclosures for the mainly zoophilic *Anopheles sinensis*, and LLINs and IRS of homes for *Anopheles anthropophagus* which prefers to feed on humans [87]. For *Anopheles dirus*, which bites during the day and rests outdoors, environmental modification was performed to eliminate breeding sites and personal protection provided to those at risk [87]. IEC/BCC activities for at-risk populations were also tailored according to the vector species [87]. In the malaria elimination stage, vector surveillance was used to characterize foci and implement appropriate vector control measures. Post-elimination, entomological surveillance is used to dynamically assess re-transmission risk based on vector density, vector species and susceptibility to insecticides, and to plan appropriate responses to imported cases and includes customs authorities to monitor vector importation at borders [87].

## Harmonization and implementation

Harmonization is crucial to ensure effective and smooth collaboration, and to facilitate learning between countries to enhance the effectiveness of their programmes. At the highest level, this is represented by the commitment of GMS Ministers of Health to eliminate malaria from the region by 2030. WHO guidance and policies are key to the development of work plans by NMPs, and are relevant for surveillance, microscopy and laboratory quality assurance, case management, and malaria elimination. However, there is no compunction for NMPs to adhere to WHO guidance or policies and it is crucial that countries are confident in the quality of WHO recommendations and perceive them to be of value in achieving their programmatic objectives.

Policy guidance for the standardization of procedures, such as laboratory methods or microscopy, must be highly specific to ensure data quality and allow data to be compared between countries. However, guidance for programmatic interventions must be flexible enough for NMPs to adapt the guidelines to their needs and objectives. For example, the WHO guidelines for malaria consider a range of artemisinin-based combinations for first-line treatment of uncomplicated malaria and do not specify the particular drugs that should be used for interventions such as MDA, TDA, or IPTf [1]. This flexibility is particularly relevant to the GMS due to the wide heterogeneity of malaria transmission patterns and the geography-specific heterogeneity in ACT effectiveness. Thus, harmonization aims must be pragmatic, appropriate, and suitable for implementation.

Changes in WHO policies are discussed with NMPs, and countries are responsible for adopting and implementing policies with technical support from WHO if required. Implementation requires considerable commitment and expertise: implementing a change to malaria treatment guidelines requires NMPs to incorporate relevant updates into their National Malaria Treatment Guidelines and work plans and secure access to the necessary commodities and resources. This involves several challenges, and barriers to implementation include funding constraints, inadequate healthcare infrastructure, a shortage of skilled health workers and bottlenecks with drug regulatory agencies and in the assured supply of diagnostics and drugs. There may also be political challenges in obtaining the necessary approvals. Establishing reliable logistics and supply chains is extremely difficult in remote areas and robust monitoring and evaluation (which often depends on reliable power and internet access) must also be in place. Particularly in the elimination setting, orders for anti-malarials and commodities may not be large enough to attract manufacturers to supply. IEC/BCC initiatives may be needed to gain community acceptance of new interventions as communities may ‘forget’ that malaria is still a risk leading them to be less accepting of interventions they feel are unwarranted or intrusive. NMPs must manage these requirements while maintaining core malaria services and adding on planned activities to accelerate elimination.

Addressing implementation barriers requires a multifaceted approach across many government agencies and partners, as well as financial investments, healthcare worker training, community engagement, and a strong political commitment to malaria control and elimination efforts. Programmes must also show great flexibility. For example, unable to obtain a quality-controlled paediatric formulation of artesunate-mefloquine, Cambodia procured and adopted paediatric artesunate-pyronaridine while maintaining artesunate-mefloquine as first-line therapy for adults with uncomplicated *P. falciparum* malaria.

Pilot implementation of new interventions in those areas with the greatest need has been a valuable approach to understand potential difficulties and to experiment with innovations. This has also enabled the development of more manageable monitoring and evaluation protocols, providing crucial evidence that can be shared across the GMS and direct potential future operational research. For example, early interventions piloting MDA for *P. falciparum* in Cambodia first, followed by Lao PDR, as part of targeted malaria elimination activities, highlighted the importance of gaining community trust to achieve sufficient coverage of mass drug administration interventions [88]. MDA was also shown to reduce *P. falciparum* malaria prevalence in Myanmar [89]. Similarly, to assess new approaches to *P. vivax* elimination, several countries are conducting pilot studies of point-of-care G6PD testing [90–92], primaquine or tafenoquine for *P. vivax* radical cure and chloroquine MDA for *P. vivax* and these experiences will be consolidated to inform programme planning.

## Conclusions

The GMS countries have achieved remarkable progress in containing artemisinin partial resistance and partner drug resistance and pushing towards malaria elimination. Malaria cases decreased by 77% and malaria deaths by 97% between 2012 and 2022. These impressive results are attributable to the unwavering commitment of the countries and the injection of substantial resources through three pivotal Global Fund grants and other sources of funding. The WHO has been in the privileged position of witnessing these achievements and supporting GMS countries where the countries identified needs and sought technical assistance for innovations, particularly for intensified approaches to accelerate malaria elimination. Historically, the GMS has been considered the global focus for anti-malarial drug resistance emergence. However, the GMS countries are writing a different future and are poised at the brink of an opportunity to take the lead in malaria elimination.

## Data Availability

Data on malaria burden are available on MME website at the weblink: https://www.who.int/initiatives/mekong-malaria-elimination-programme. Data on drug efficacy are available on the WHO Threat Maps at the weblink: https://apps.who.int/malaria/maps/threats/.

## Abbreviations

ARCE: Artemisinin Resistance Containment and Elimination
ACT: Artemisinin-based combination therapy
BCC: Behaviour change communication
BMGF: Bill and Melinda Gates Foundation
ERAR: Emergency Response to Artemisinin Resistance
Global Fund: Global Fund to Fight AIDS, Tuberculosis and Malaria
GMS: Greater Mekong Subregion
GPARC: Global Plan for Artemisinin Containment
iDES: Integrated drug efficacy surveillance
IEC: Information education communication
IPTf: Intermittent preventive treatment for forest goers
IRS: Indoor residual spraying
Lao PDR: Lao People’s Democratic Republic
LLIHN: Long-lasting insecticide-treated hammock net
LLIN: Long-lasting insecticide-treated net
MDA: Mass drug administration
MEDB: Malaria Elimination Database
MME: Mekong Malaria Elimination
MMP: Mobile and migrant populations
NMP: National Malaria Programme
RAI: Regional artemisinin-resistance initiative
TDA: Targeted Drug Administration
TES: Therapeutic efficacy studies
USAID-PMI: U.S. Agency for International Development President’s Malaria Initiative
VMW: Village malaria worker
WHO: World Health Organization
