# Publisher Correction: Progress towards malaria elimination in the Greater Mekong Subregion: perspectives from the World Health Organization

**DOI:** 10.1186/s12936-024-04943-w

**Published:** 2024-04-25

**Authors:** Giulia Manzoni, Rady Try, Jean Olivier Guintran, Céline Christiansen-Jucht, Elodie Jacoby, Siv Sovannaroth, Zaixing Zhang, Vilasack Banouvong, Matthew Scott Shortus, Rita Reyburn, Chitsavang Chanthavisouk, Nay Yi Yi Linn, Badri Thapa, San Kyawt Khine, Prayuth Sudathip, Deyer Gopinath, Nguyen Quang Thieu, Mya Sapal Ngon, Dai Tran Cong, Liu Hui, James Kelley, Neena Nee Kesar Valecha, Maria Dorina Bustos, Charlotte Rasmussen, Luciano Tuseo

**Affiliations:** 1 WHO Mekong Malaria Elimination Programme, Phnom Penh, Cambodia; 2 World Health Organization Country Office, Phnom Penh, Cambodia; 3 National Center for Parasitology, Entomology and Malaria Control, Phnom Penh, Cambodia; 4 Centre for Malariology, Parasitology and Entomology, Vientiane, Lao PDR; 5 World Health Organization Country Office, Vientiane, Lao PDR; 6 National Malaria Control Programme, Nay Pyi Taw, Myanmar; 7 World Health Organization Country Office, Yangon, Myanmar; 8 Division of Vector Borne Diseases, Department of Disease Control, Bangkok, Thailand; 9 World Health Organization Country Office, Bangkok, Thailand; 10 National Institute of Malariology, Parasitology and Entomology, Hanoi, Viet Nam; 11 World Health Organization, Hanoi, Viet Nam; 12 Yunnan Institute of Parasitic Diseases, Yunnan, China; 13 World Health Organization, Regional Office for the Western Pacific, Manila, Philippines; 14 World Health Organization, Regional Office for South-East Asia, New Delhi, India; 15 Global Malaria Programme, World Health Organization, Geneva, Switzerland; 16 Independent Consultant, Antananarivo, Madagascar; 17 Independent Consultant, Le Bar Sur Loup, France; 18 Independent Consultant, Ho Chi Minh, Viet Nam

**Correction: Malaria Journal (2024) 23:64** 10.1186/s12936-024-04851-z

Following publication of the original article [1], it was brought to the journal’s attention that the article had published with the Creative Commons BY 4.0 license, whereas the correct license is the Creative Commons BY 3.0 IGO license. The license has since been corrected in the article. The publisher thanks you for reading this erratum and apologizes for any inconvenience caused.
